# Molecular Imaging of Activated Platelets Allows the Detection of Pulmonary Embolism with Magnetic Resonance Imaging

**DOI:** 10.1038/srep25044

**Published:** 2016-05-03

**Authors:** Timo Heidt, Simon Ehrismann, Jan-Bernd Hövener, Irene Neudorfer, Ingo Hilgendorf, Marco Reisert, Christoph E. Hagemeyer, Andreas Zirlik, Jochen Reinöhl, Christoph Bode, Karlheinz Peter, Dominik von Elverfeldt, Constantin von zur Muhlen

**Affiliations:** 1 Department of Cardiology and Angiology I, Heart Center Freiburg University, Germany; 2 Department of Radiology, Medical Physics, Medical Center – University of Freiburg, Faculty of Medicine, Germany; 3 German Cancer Consortium (DKTK), Partner Site Freiburg, Germany; 4 Australian Centre for Blood Diseases, Monash University, Victoria, Australia; 5 Baker IDI Heart and Diabetes Institute, Melbourne, Australia

## Abstract

Early and reliable detection of pulmonary embolism (PE) is critical for improving patient morbidity and mortality. The desire for low-threshold screening for pulmonary embolism is contradicted by unfavorable radiation of currently used computed tomography or nuclear techniques, while standard magnetic resonance imaging still struggles to provide sufficient diagnostic sensitivity in the lung. In this study we evaluate a molecular-targeted contrast agent against activated platelets for non-invasive detection of murine pulmonary thromboembolism using magnetic resonance imaging. By intravenous injection of human thrombin, pulmonary thromboembolism were consistently induced as confirmed by immunohistochemistry of the lung. Magnetic resonance imaging after thrombin injection showed local tissue edema in 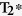 weighted images which co-localized with the histological presence of pulmonary thromboembolism. Furthermore, injection of a functionalized contrast agent targeting activated platelets provided sensitive evidence of focal accumulation of activated platelets within the edematous area, which, *ex vivo*, correlated well with the size of the pulmonary embolism. In summary, we here show delivery and specific binding of a functionalized molecular contrast agent against activated platelets for targeting pulmonary thromboembolism. Going forward, molecular imaging may provide new opportunities to increase sensitivity of magnetic resonance imaging for detection of pulmonary embolism.

Pulmonary thromboembolism (PE) is a mayor burden of morbidity and mortality worldwide, especially, if detected late or not at all^1^,^2^. With an annual incidence of 100 to 200 cases per 100 000, venous thromboembolism constitutes the third most frequent cardiovascular disease in the Western world^3^,^4^.

Standard techniques for detection of pulmonary embolism involve contrast-enhanced computed tomography or ventilation-perfusion szintigraphy^5^,^6^. Both techniques have proven high sensitivity and specificity for detection of pulmonary embolism, but involve radiation, which is unfavorable in young patients or if the pre-test probability is low.

Although magnetic resonance imaging (MRI) would provide a radiation-free alternative to these conventional techniques^7^, susceptibility artifacts in the lung due to repeated tissue-air transitions challenge image quality and limit sensitivity especially for the detection of smaller thromboemboli^8^,^9^. As a consequence, MRI is not recommended for detection of pulmonary embolism by current US or European guidelines^10^,^11^.

Molecular imaging with target specific imaging probes constitutes an emerging field because of its excellent possibilities to facilitate highly selective detection of epitopes even at very low density or in sparse contrast regions. Thromboembolism are formed from a mixture of fibrin, coagulation factor-mediated peptides and platelets. Among these, fibrin and alpha_2_-antiplasmin have previously been targeted in diverse formulations using gadolinium or perfluorocarbon compounds for contrast imaging and have shown promising results for the detection of venous thromboembolism^12^,^13^,^14^. With EP-2104R, fibrin-targeting has translated from bench to bedside, but a routine clinical application is still pending^15^.

Targeting platelets might be an additional strategy for the detection thromboembolism. We have previously described a single-chain antibody directed against the highly abundant ligand induced binding sites (LIBS) of the GPIIb/IIIa receptor which are specific for activated platelets^16^,^17^. Conjugated to microparticles of iron oxide (MPIO), this contrast agent has successfully been used for MRI-based detection of platelet activation in carotid thrombosis^18^.

In this study we investigate if targeting of activated platelets using MPIO-labeled single-chain antibodies can be used for the detection of murine pulmonary thromboembolism with magnetic resonance imaging.

## Results

**Figure 1:**
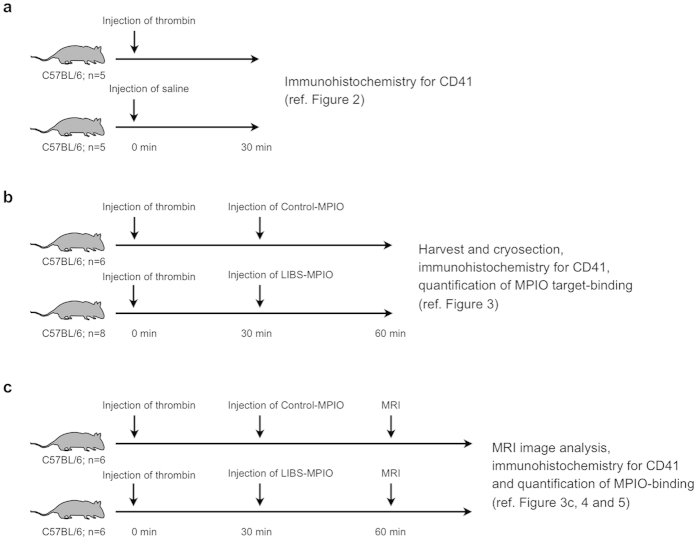
Experimental setup. Experiments performed in the study were conducted in three experimental cohorts. (**a**) Experimental setup for data shown in Fig. 2. Lungs of mice intravenously injected with either thrombin or saline were harvested 30 minutes after injection. (**b**) Experimental setup for data shown in Fig. 3. Mice were intravenously injected with thrombin. 30 minutes after injection, mice received LIBS-MPIO or Control-MPIO contrast agent. After additional 30 min lungs were harvested. (**c**) Experimental setup for data shown in Figs 3c, 4 and 5. Mice received thrombin and after 30 minutes LIBS-MPIO or Control-MPIO contrast agent. Additional 30 minutes later magnetic resonance imaging was conducted. Directly after magnetic resonance imaging lungs were harvested for *ex vivo* analysis.

Experiments performed in the study were conducted in three experimental cohorts as specified in Fig. 1.

### Intravenous injection of human thrombin induces pulmonary thromboembolism

**Figure 2:**
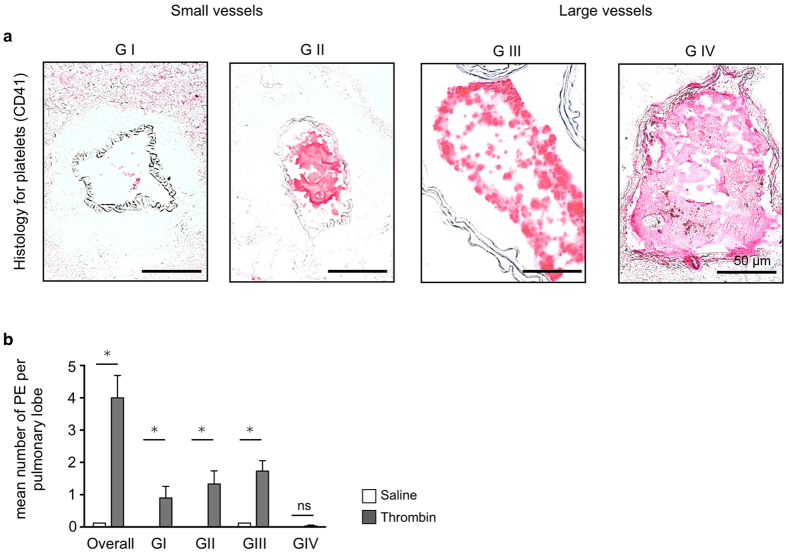
Grading of thrombin-induced pulmonary thromboembolism. (**a**) Grading of pulmonary embolism according to vessel size and degree of vessel obstruction (GI-IV). Small vessel (maximum diameter <150 μm: GI < 50% vessel obstruction, GII > 50% vessel obstruction) or large vessels (maximum diameter >150 μm: GIII < 50% vessel obstruction, GIV > 50% vessel obstruction). Bar indicates 50 μm. (**b**) Mean number of pulmonary embolism per lobe after intravenous thrombin injection, overall and according to embolism grade compared to saline injection (n = 5 mice per group, Data are shown as mean ± s.e.m.; p < 0.05, Mann-Whitney U Test; ns = not significant).

To induce pulmonary embolism human thrombin was injected intravenously using a tail vein catheter. Slow injection ensured survival of approximately 80% of study animals (data not shown). Immunohistochemistry for the platelet-specific epitope CD41 was used to confirm thromboemboli in pulmonary vessels. Based on vessel size and degree of vessel obstruction pulmonary thromboembolism were graded in four different size categories (Fig. 2a). Thromboembolism with grades I through III were most frequent, while the incidence of very large pulmonary thromboembolism (grade IV) was rare. In comparison, almost no thrombosis was found in pulmonary vessels of saline injected animals (Fig. 2b).

### LIBS-MPIO selectively binds to activated platelets in pulmonary thromboembolism

**Figure 3:**
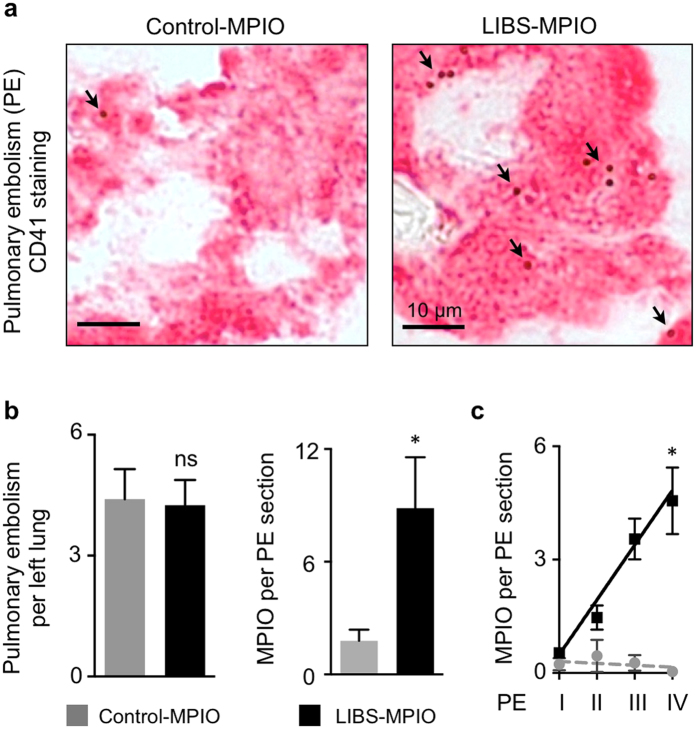
LIBS-MPIO contrast agent specifically binds to activated platelets of pulmonary thromboembolism. (**a**) Immunohistochemistry staining for platelet CD41 in thrombin-induced pulmonary embolism (PE) after injection of LIBS-MPIO or Control-MPIO. ↘ MPIO visible as small spheres on the surface of CD41^+^ cells. Bar indicates 10 μm. (**b**) Number of PE per left lung (left) after injection of human thrombin and LIBS-MPIO compared to thrombin and Control-MPIO Quantification of mean MPIO-binding per PE section (right) was performed by counting MPIO co-localized to CD41^+^ cells using light microscopy with 63x to 100x magnification. (n = 6–8 mice per group, p < 0.05; ns = not significant, Mann-Whitney U Test). (**c**) Correlation of PE grade with number of MPIOs bound to PE section after injection of LIBS-MPIO or Control-MPIO (n = 6 mice per group, p < 0.05, Pearson correlation). Data are shown as mean ± s.e.m.

We next applied LIBS-MPIO or Control-MPIO after 30 minutes following injection of human thrombin. Figure 3a shows activated platelets in pulmonary embolism and co-localization with presence of contrast agent (MPIO) after injection of labeled LIBS or Control single-chain antibody. At comparable amounts of pulmonary embolism after injection of human thrombin, LIBS contrast agent resulted in significantly higher MPIO target binding on thromboembolism compared to Control-MPIO (n = 6–8 mice per group, p < 0.05; Mann-Whitney U Test; Fig. 3b). Comparison of MPIO target binding to thromboembolism grade shows significant correlation for LIBS-MPIO (R^2^ = 0.94) compared to Control-MPIO (R^2^ = 0.34; n = 6 mice per group, p < 0.05, Pearson correlation; Fig. 3c).

### Injection of human thrombin focally increases pulmonary signal in 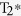 - weighted MRI

**Figure 4:**
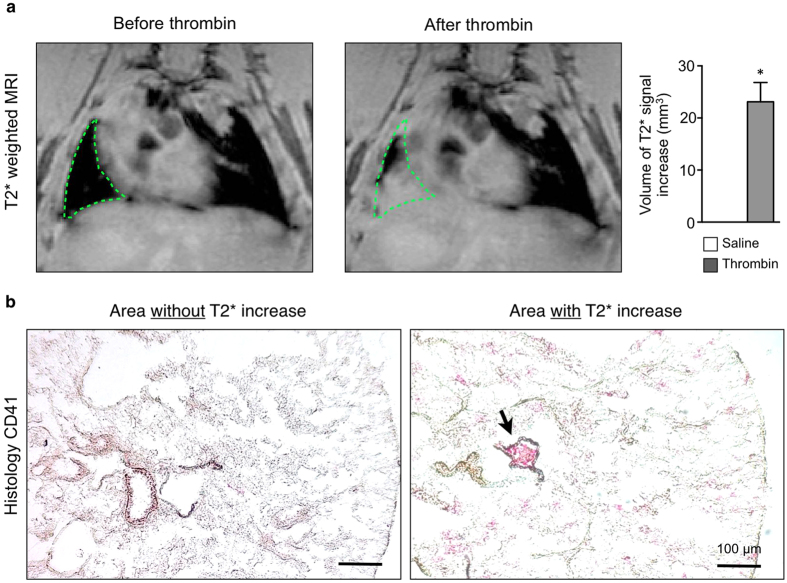
Thrombin-induced pulmonary edema correlates with presence of thromboembolism. (**a**) *In vivo* 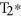 weighted MRI of the thorax before (left) and after (right) intravenous injection of human thrombin. Green dotted line indicates area of 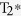 signal increase. Compared to saline injection, injection of human thrombin induced significant focal signal increase in 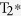 weighted images (saline n = 4-vs. thrombin n = 12 mice per group, p < 0.05, Mann-Whitney U Test). (**b**) Immunohistochemistry staining for platelet CD41 (20x magnification) in thrombin-induced pulmonary embolism (PE). Bar indicates 100 μm. Relevant pulmonary embolism can be found in areas with increased 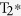, but is absent in tissue without 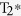 signal increase. Data are shown as mean ± s.e.m.

Performing *in vivo* 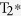 weighted MRI in mice before and after injection of human thrombin, we found focal increase of 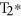 signal in 75% of thrombin injected animals as signs of segmental tissue edema around and subsequent of the obstructed vessel after thrombin injection. Of note, signal increase was not present in saline injected animals (Fig. 4a). Histology for platelet CD41 from resected lungs revealed that pulmonary embolism (GII-IV) correlated with areas of increased 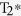 signal (Fig. 4b), while only minor thromboembolism (GI) was found in areas without 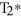 increase.

### MPIO contrast agent deposition can be detected within 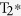 positive segmental edema

**Figure 5:**
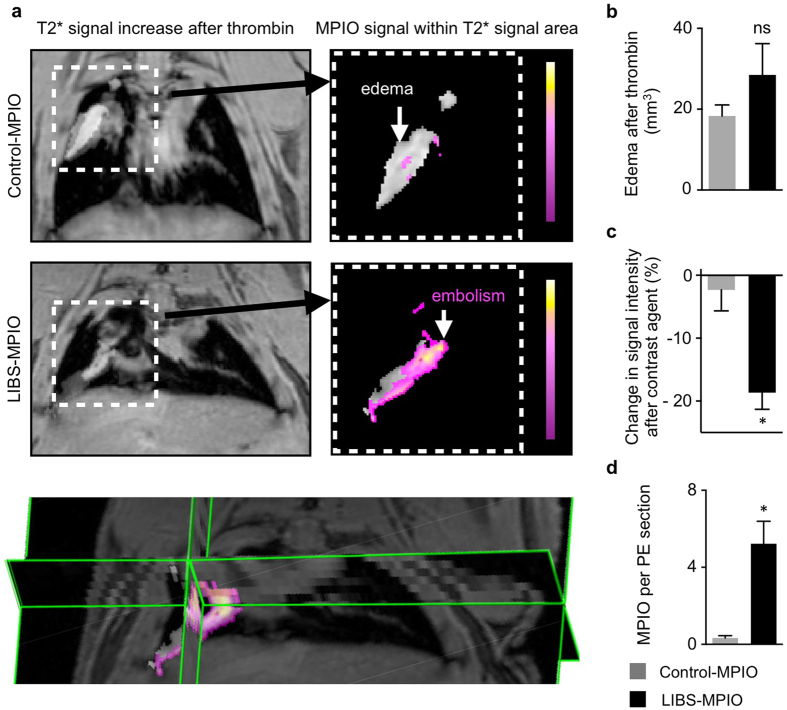
Contrast agent-induced signal voids can be detected within edematous area. (**a**) *In vivo* 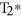 weighted MRI of the thorax after intravenous application of human thrombin (left panel) and injection of Control-MPIO (right panel, above) or LIBS-MPIO (right panel below). Right panel show extracted overlay of edema (gray zone) and MPIO-induced signal decay within this area (pseudo-colored pink-yellow). Below, 3D map of edema after thrombin and corresponding signal overlay after injection of LIBS-MPIO in the murine lung. (**b**) Edema area after thrombin injection in LIBS-MPIO or Control-MPIO group does not differ (6 mice per group, ns = not significant). (**c**) Percent change of signal intensity in human thrombin-induced edema after injection of LIBS-MPIO or Control-MPIO (n = 6 mice per group, p < 0.05, Mann-Whitney U Test). (**d**) Mean number of MPIO found per immunohistochemistry section of pulmonary embolism after *ex vivo* preparation and staining for platelet CD41 (n = 6 per group, p < 0.05, Mann-Whitney U Test). Data are shown as mean ± s.e.m.

After injection of human thrombin and acquisition of 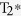 weighted images, LIBS-MPIO or Control-MPIO contrast agent were injected intravenously. Injection of LIBS-MPIO was followed by a significant signal loss at the border of the edema compared to signal loss after injection of Control-MPIO (n = 6 mice per group, p < 0.05, Mann-Whitney U Test; Fig. 5a–c). Increased numbers of MPIO found in corresponding thromboembolism reflected this difference (n = 6 mice per group, p < 0.05, Mann-Whitney U Test; Fig. 5d).

## Discussion

Pulmonary embolism is a major health burden responsible for considerable morbidity and mortality also in young patients. In the present study we applied a molecular-targeted contrast agent (LIBS-MPIO) towards accumulation of activated platelets for highly sensitive, non-invasive detection of pulmonary embolism using magnetic resonance imaging in a murine model. Injection of human thrombin via a tail vein catheter *in vivo* resulted in formation of local pulmonary edema as described by gain of signal in 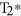 weighted MR images. Microparticles of iron oxide coupled to LIBS-single-chain antibody allowed for non-invasive detection due to a loss of signal within the edema while binding to activated platelets. *Ex vivo*, this was confirmed by showing specific binding of LIBS-MPIO to activated platelets. Histology showed the presence of thromboembolism within the representative pulmonary edema as well as specific deposition of microparticles of iron-oxide at the target of interest.

Molecular imaging for the detection of pulmonary embolism with magnetic resonance imaging has mainly focused on fibrin^13^,^15^,^19^,^20^. Besides fibrin, platelets have been shown to be another relevant component of PE^21^. The susceptibility to activation and tendency towards accumulation - once stimulated by other platelets, fibrin or clotting factors - makes platelets an ideal target for the detection of very small amounts of substrate.

Furthermore, besides aggregation, platelets also release vasoactive agents such as serotonin, prostaglandins and thromboxane A2 which drive adverse vasoconstriction and pulmonary hypertension^22^. Additionally, microparticles from activated platelets are involved in vascular inflammation and coagulation^23^. Platelets thus are important not only for vessel obstruction, but also for subsequent vascular inflammation and pathogenesis following pulmonary embolism. Looking for alternative fields of application next to imaging pulmonary embolism, targeting and imaging of activated platelets in the lung using magnetic resonance imaging could also be explored for imaging in pulmonary hypertension, pulmonary vascular inflammation and recurrent thrombotic vessel obstruction, too. To this end, we have previously demonstrated that targeting of activated platelets could be used for the detection of cerebrovascular or myocardial inflammation^24^,^25^.

Pulmonary embolism was induced using a tail vein catheter for injection of human thrombin, as previously described by Nagaschima *et al*.^26^ Compared to alternative vascular access routes, e.g. the jugular vein^27^, the remote location of the tail vein as compared to the lung is less likely to induce susceptibility artifacts. Furthermore, injection of human thrombin via the tail vein allowed for repetitive MR image acquisition before and after induction of pulmonary embolism without moving the animal in the MR scanner, thus reducing motion artifacts and providing excellent in-plane consistency of acquired images.

Injection of human thrombin resulted in pulmonary thrombi of different sizes as confirmed by immunohistochemistry for platelet CD41. Mortality rate after injection of human thrombin was about 20%, well in line with previous reports^14^,^26^. Mortality is most likely due to the size of the PE with central vessel obstruction leading to fatal right-sided heart failure. This may explain why grade grade IV pulmonary embolism was a rare event in this experimental setup. As the lung is a highly vascularized organ, *ex vivo* histology of these mice for detection of such a fatal pulmonary thromboembolism failed due to post-mortem clotting artifacts. Comparing results after human thrombin injection to animals injected with saline, however, confirmed specificity of the human-thrombin approach for pulmonary embolism grades G II-IV.

Microparticles of iron oxide (MPIO) exert a very strong signal in magnetic fields surpassing their actual particle size approximately 50 fold, which allows for detection of very sparse target substrate. Visibility of MPIOs by light microscopy additionally facilitates detection and quantification of contrast agent binding *ex vivo*, as well as correlation with findings in MRI. Quantification of MPIOs per field of view in pulmonary embolism revealed a good correlation with embolism size. In a translational application, this would allow for non-invasive sizing and mapping of pulmonary embolism dissemination. Larger scale studies would need to evaluate if this information could be used as a prognostic marker.

A relevant limitation of the contrast agent used is that MPIOs impose their contrast as negative signal, caused by field disturbance of iron oxide particles and resulting in local signal loss in the area of contrast deposition^28^. Furthermore, the lung is a problematic organ for magnetic resonance imaging with 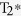 due to the high degree of phase transition between tissue and air also causing susceptibility artifacts. Therefore, despite the excellent sensitivity, MPIOs may not seem ideal for application in the lung, at first sight.

In this model, injection of human thrombin caused an early and persistent, segmental signal gain in T_2_ and 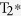 weighted images comparable to zones of pulmonary edema observed in humans in the setting of post-embolic pulmonary infarction^29^,^30^. Histology of the edematous pulmonary segments revealed presence of thromboembolism in the corresponding area, while no relevant thrombus was observed in the absence of pulmonary edema. After injection of LIBS-MPIO contrast agent, the signal gain induced by the edema provided a background which allowed for sensitive detection of accumulation of activated platelets at the border of edematous zones demarcating the pulmonary embolism as focal zones of signal loss within the edema. While this phenomenon allowed for the use of MPIOs for target-contrast, smaller pulmonary embolism may not cause relevant pulmonary edema and therefore could escape detection despite proper target binding. Also, in humans, the presence of pulmonary edema is mostly seen in massive embolism which is challenging the accuracy of detection of smaller thromboembolism. This may explain why *in vivo* the correlation between MRI signal and embolism size remains challenging notwithstanding promising *ex vivo* results.

A further limitation is that analyzing signal decay within a defined area with previous gain of signal requires that the signal loss reported refers to the entire edematous zone, instead of the thromboembolism only. Therefore, the local signal reported is most likely to be underestimated and true local changes might be more pronounced.

Taking these limitations into account, this murine study provided evidence that platelets are a suitable target for detection of pulmonary embolism with molecular imaging. Animal models with less edema formation, which might be caused by missing collateralization of the pulmonary circulation in murine models, will need to confirm the obtained data. Furthermore, evaluation of positive contrast agents as has been shown for gadolinium could overcome the technical need for background edema^13^. Alternatively, accumulation of 19-Fluor in vascular thrombosis has been shown to give sufficient signal for non-invasive detection with MRI^14^. Also, biocompatible magnetoliposomes loaded with contrast agent could overcome toxicity concerns of MPIOs^31^. Ultimately, transfer studies involving human patients will have to show if sensitivity and specificity of this approach is suitable for future clinical applications.

## Conclusions

To our knowledge, this is the first study investigating the detection of pulmonary embolism via targeting activated platelets with molecular magnetic resonance imaging. The involvement of activated platelets in formation of thromboemboli by aggregation as well as pulmonary vascular pathophysiology via platelet-induced vascular inflammation, makes selective imaging of activated platelets an attractive target with possible future application in clinical diagnostics as well as basic research on pulmonary vascular disease. In this study we show that activated platelets in pulmonary embolism can be detected non-invasively by magnetic resonance imaging. Persistent limitations request further studies and evaluation of different MR-suitable contrast agents to evaluate its use for future clinical application. Radiation-free assessment of pulmonary embolism combined with anatomical and functional information would add great value as compared to existing computed tomography or ventilation-perfusion scintigraphy and might be helpful for the physician in guiding the patient.

## Methods

### Animals

Female C57BL/6N mice, age 10–12 weeks, were purchased from Charles River Laboratories (Sulzfeld, Germany). Mice were housed in cages of 5 animals. Chow and water was provided without restriction. The numbers of animals used for each study group are indicated in the figure legends. All experimental protocol were approved by the ethics committee of Freiburg University and the regional council of Freiburg, Baden-Wuerttemberg, Germany: licence number 35-9185.81/G-13/120). Experiments were conducted in accordance with FELASA, GV-SOLAS standards for animal welfare.

### Model of murine thrombin-induced pulmonary embolism

Pulmonary embolism was induced as previously described by Nagaschima *et al*.^26^ Prior to the induction of pulmonary embolism, mice were anesthetized by subcutaneous injection of a mixture containing ketamine (250 mg/kg bw) and xylazine (12.5 mg/kg bw). Thereafter, a tail vein catheter was placed. Human thrombin (Thromborel S, Bayer, Germany) was slowly injected in a total volume of 100 μl saline at a final concentration of 2500 Units per kg bw. Thrombin injection resulted in an approximate survival rate of 80%.

### Platelet specific single-chain antibody (LIBS-MPIO)

Monoclonal anti-LIBS single-chain antibody selectively binds to ligand-induced binding sites which are exposed on the activated glycoprotein IIb/IIIa receptor, but are not exposed on resting platelets^32^. Single-chain antibody cloning, production and purification was performed as previously described^33^. A non-binging control single-chain antibody was constructed by amino base exchange. For MPIO-labeling, cobalt-functionalized auto-fluorescent MPIOs with a diameter of 1 μm were conjugated to the histidine-tag of the LIBS/control single-chain antibody according to the manufacturer’s instruction (Dynal Biotech, Oslo, Norway). LIBS-single-chain antibody conjugated to MPIO will be called “LIBS-MPIO” contrast agent. Conjugation to unspecific control single-chain antibody will be referred to as “Control-MPIO” contrast agent.

### Contrast agent injection

Contrast agents were injected at 0.5 mg/kg body weight in 100 μl PBS using a tail vein catheter (80 cm length), 30 minutes after induction of pulmonary embolism to ensure enough time for thrombus formation. Imaging was performed 30 minutes after contrast agent injection to reduce unspecific binding properties and increase specificity.

### Magnetic Resonance Imaging

All magnetic resonance experiments were performed on a dedicated small animal MRI system (BioSpec94/20, Bruker Germany), run with AVANCE III electronics, Paravision 5.1 software, and employing a quadrature whole body mouse birdcage resonator (id: 36 mm).

After induction of anesthesia and catheter placement in the tail vein, animals were placed head first in supine position onto the animal bed with a breathing sensor pad beneath the animal. Animal temperature was maintained by warm-water supported heating of the animal bed. During MRI, anesthesia was maintained via isoflurane in O_2_ in a range of 0,8–1.8 Vol%, stabilizing the animals at a breathing rate of approximately 70 breaths per minute.

Imaging the lung all MRI protocols where retrospectively gated Intragate-FLASH sequences in order to maintain a breathing-rate independent signal steady state and therefore a constant image contrast.

A multislice pilot scan was performed in order to verify the animal position and to plan the main sequences. A 2D multislice Intragate FLASH covering the complete lung, once in coronal and once in axial orientation was run three times. The scans where repeated once as a baseline, once after administration of thrombin, and once after administration of LIBS-MPIO. The intragate scans had a TE/TR of 1.39 ms/106 ms, 50 repetitions, a flip angle of 15°, a resolution of 113 μm in plane at a slice thickness of 400 μm, and a total acquisition time of 11′22′′ per scan.

### Image Analysis

Analysis of the images that were acquired (1) native, (2) 10 min post thrombin injection and (3) 30 min post LIBS-MPIO or LIBS-Control agent injection was performed using a custom-made software (Matlab, The Mathworks, USA). To compensate for motion and misalignment that occurred over the time span of the scans (120 min), each slice in datasets 1–3 was subjected to a non-rigid transformation to match a slice that was the sum of all three.

Edema was defined in the post-thrombin images (2) by a gain in signal of more than 50% with respect to the naive image for groups of ten or more connected voxels in each slice. The volume of the edema was quantified by counting these voxels across all slices. Areas clearly outside of the lung that showed an increased signal i.e. due to motion artifacts or misalignments were discarded manually.

Within the area of an edema, the effect of the contrast agent was quantified by the total change in signal, i.e. the sum of signal intensities within the edema in post-thrombin images (2) compared to the sum in post-agent images (3).

For presentation, the edema area as well as the color-coded change in signal was superimposed on the anatomical images post-thrombin (2).

### Preparation of tissue for cryosection

For tissue preparation, mice where perfused with 10 ml of PBS in deep anesthesia via the right ventricle to clear pulmonary vessels from remnant blood. Afterwards Tissue-Tek embedding medium OCT (Sakura Finetek, Netherlands) diluted 1:3 parts with PBS was instilled into lungs via tracheal injection. Lungs where fully embedded in OCT and shock-frozen on dry ice. Tissue blocks where sectioned with 10 μm thickness at 30 μm intervals and fixed using acetone.

### Histology

Platelets were stained using a rat anti-mouse CD41 antibody (GTX 76011, GeneTex, Irvine/CA, USA) or unspecific control IgG1 isotype (MCA1211, Serotec, Puchheim, Germany). For secondary staining a biotinylated rabbit anti-rat IgG (BA-4001, Vector, Burlingame/CA, USA) was used. Afterwards, alkaline phosphatase and substrate kit (AK-5000 & SK-5100; Vector, Burlingame/CA, USA) after levamisol pre-treatment (X3021, DAKO, Hamburg, Germany) was applied for antibody detection. Sections were then embedded in Kaiser’s Glyceringelatine (1092420100, Merck, Hamburg, Germany). Due to their size (1 μm), MPIO beads are visible using 63x magnification without further staining. MPIOs were quantified as mean number of MPIOs of 3 sections per section at 63x magnification.

Histology sections were analyzed using a light microscope (Zeiss optics, Germany). Pulmonary embolism was quantified as mean of 3 representative sections of the according. Pulmonary emboli were graded according to size of obstructed vessels: small vessel (maximum diameter < 150 μm: GI < 50% vessel obstruction, GII > 50% vessel obstruction) or large vessels (maximum diameter > 150 μm: GIII < 50% vessel obstruction, GIV > 50% vessel obstruction).

### Statistics

Statistical analyses were supported by GraphPad Prism software (GraphPad Software, Inc.). Results are depicted as mean ± standard error of mean if not stated otherwise. For two-group comparison, students t-test was applied if the pre-test for normality (D’Agostino-Pearson normality test) and equality of variances (Bartlett’s test) was not rejected at 0.05 significance level, otherwise a Mann-Whitney U test for nonparametric data was used. For a comparison of more than two groups an ANOVA, followed by a Bonferroni test for multiple comparison, was applied.

P values of p < 0.05 indicate statistical significance.

## Additional Information

**How to cite this article**: Heidt, T. *et al*. Molecular Imaging of Activated Platelets Allows the Detection of Pulmonary Embolism with Magnetic Resonance Imaging. *Sci. Rep*. **6**, 25044; doi: 10.1038/srep25044 (2016).
